# Autonomous and controlled motivational regulations for multiple health-related behaviors: between- and within-participants analyses

**DOI:** 10.1080/21642850.2014.912945

**Published:** 2014-04-30

**Authors:** M.S. Hagger, S.J. Hardcastle, A. Chater, C. Mallett, S. Pal, N.L.D. Chatzisarantis

**Affiliations:** ^a^ Health Psychology and Behavioral Medicine Research Group, School of Psychology and Speech Pathology, Curtin University, Perth, Australia; ^b^ School of Sport and Service Management, University of Brighton, Eastbourne, UK; ^c^ Department of Practice and Policy, School of Pharmacy, University College London, London, UK; ^d^ School of Human Movement Studies, University of Queensland, Brisbane, Australia; ^e^ School of Public Health, Curtin University, Perth, Australia

**Keywords:** self-determination theory, intentions, causality orientations, dispositional motivation, self-regulation

## Abstract

Self-determination theory has been applied to the prediction of a number of health-related behaviors with self-determined or *autonomous* forms of motivation generally more effective in predicting health behavior than non-self-determined or *controlled* forms. Research has been confined to examining the motivational predictors in single health behaviors rather than comparing effects across multiple behaviors. The present study addressed this gap in the literature by testing the relative contribution of autonomous and controlling motivation to the prediction of a large number of health-related behaviors, and examining individual differences in self-determined motivation as a moderator of the effects of autonomous and controlling motivation on health behavior. Participants were undergraduate students (*N* = 140) who completed measures of autonomous and controlled motivational regulations and behavioral intention for 20 health-related behaviors at an initial occasion with follow-up behavioral measures taken four weeks later. Path analysis was used to test a process model for each behavior in which motivational regulations predicted behavior mediated by intentions. Some minor idiosyncratic findings aside, between-participants analyses revealed significant effects for autonomous motivational regulations on intentions and behavior across the 20 behaviors. Effects for controlled motivation on intentions and behavior were relatively modest by comparison. Intentions mediated the effect of autonomous motivation on behavior. Within-participants analyses were used to segregate the sample into individuals who based their intentions on autonomous motivation (autonomy-oriented) and controlled motivation (control-oriented). Replicating the between-participants path analyses for the process model in the autonomy- and control-oriented samples did not alter the relative effects of the motivational orientations on intention and behavior. Results provide evidence for consistent effects of autonomous motivation on intentions and behavior across multiple health-related behaviors with little evidence of moderation by individual differences. Findings have implications for the generalizability of proposed effects in self-determination theory and intentions as a mediator of distal motivational factors on health-related behavior.

## 1. Background

### 1.1. Health-related behaviors and motivation

Many chronic illnesses and conditions in developed nations have, directly or indirectly, behavioral roots. Examples of health problems linked to behavior abound and include cardiovascular disease, obesity, diabetes, certain cancers (e.g. lung cancer, skin cancer), and sexually transmitted infections (Daar et al., 2007; World Health Organization, 2008). Health behaviors such as physical activity, drinking less alcohol, following a healthy diet, smoking cessation, and using barrier contraception have been identified as behaviors that may lead to a protective effect from specific or multiple conditions (Beaglehole et al., 2011; Hagger, Wood, Stiff, & Chatzisarantis, 2009; Spring, Moller, & Coons, 2012; Yang, Yang, Zhu, & Qiu, 2011). The preponderance of these health conditions and the effectiveness of health-related behavior in conferring reduced risk have catalyzed research into the environmental and psychological factors that affect individuals' uptake and maintenance of health-related behavior (Sallis, 2010). As manipulating and changing an individual's environment are extremely costly (e.g. subsidizing health foods or providing condoms) or unpopular (e.g. increasing alcohol costs), there is considerable interest in the factors that affect individuals' *self-regulation*, that is, their capacity to make and maintain changes to their behavior in the absence of external prompting, incentive, or reinforcement (De Ridder & De Wit, 2006; Hagger, 2010).

Health psychologists and interventionists interested in behavioral solutions to health issues have applied social psychological theories to identify the psychological factors associated with health-related behavior and the processes and mechanisms involved (Hagger & Luszczynska, 2014; Hagger & Hardcastle, 2014; Lippke, Nigg, & Maddock, 2012; Michie, Rothman, & Sheeran, 2007). Such an endeavor may yield an evidence base for psychological constructs that are viable targets for behavior-change interventions (Dombrowski et al., 2012; Michie & Johnston, 2012; Michie & West, 2013; Stavri & Michie, 2012).

### 1.2. Self-determination theory

Self-determination theory is a key theory of motivation that has made a substantial contribution to predicting self-regulated behavior, including numerous health-related behaviors (Deci & Ryan, 1985b, 2000). The theory suggests that the quality of individuals' motivation affects the extent to which individuals will engage in, and persist with, behaviors (Deci & Ryan, 1985b, 2000). Central to the theory is the distinction between two forms of motivation: autonomous and controlled. The forms of motivation reflect individuals' rationale or reasons for engaging in tasks and are driven by perceptions as to whether the behavior will service an individual's psychological needs. Autonomous motivation is defined as engaging in a behavior because it is perceived to be consistent with intrinsic goals or outcomes and emanates from the self. In other words, the behavior is *self*-determined. Individuals engaging in behaviors feel a sense of choice, personal endorsement, interest, and satisfaction and, as a consequence, are likely to persist with the behavior. The behavior is consistent with and supports the individuals' innate needs for autonomy, the need to feel like a personal agent in one's environment, competence, and the need to experience a sense of control and efficacy in one's actions. Individuals acting for autonomous reasons are more likely to initiate and persist with a behavior without any external reinforcement and contingency. Autonomously motivated individuals are, therefore, more likely to be effective in self-regulation of behavior. Controlled motivation, in contrast, reflects engaging in behaviors for externally referenced reasons such as to gain rewards or perceived approval from others or to avoid punishment or feelings of guilt. Individuals engaging in behavior for controlled reasons feel a sense of obligation and pressure when engaging in the behavior and are only likely to persist with the behavior as long as the external contingency is present. If the reinforcing agent is removed, action is likely to desist. Individuals who are control-motivated are therefore less likely to be self-regulated. The behavior is not perceived as supporting psychological needs and is instead likely to be viewed as need-thwarting.

Self-determination theory proposes a more nuanced differentiation of the autonomous and controlled forms of motivation underpinning action. Ryan and Connell (1989) developed a taxonomy of motivational *regulations* known as the perceived locus of causality. The taxonomy was conceptualized as akin to a continuum ranging from the most autonomous to the most controlling forms. Intrinsic motivation was identified as the prototypical form of autonomous motivation, reflecting motives for engaging in behavior for the inherent interest and satisfaction derived from engaging in the action itself. Identified regulation, a form of autonomous motivation, was situated immediately adjacent to intrinsic motivation on the continuum. Identified regulation reflects engaging in a behavior for personally relevant outcomes that are important to the individual's sense of self rather than for the inherent interest derived from engaging in the behavior itself. Although identified regulation reflects engaging in behaviors for reasons separate from the behavior itself, both are conceived as autonomous. External regulation represents the prototypical form of control regulation and reflects engaging in actions for external reinforcement such as gaining a reward or avoiding punishment. Adjacent to external regulation on the continuum lies introjected regulation. This reflects engaging in behaviors to avoid an internally perceived but externally referenced contingency such as avoiding guilt or shame or gaining approval or contingent self-worth. Although the perceived locus of causality is conceived as a continuum, research has demonstrated that a profile approach toward the taxonomy is perhaps more effective and better characterizes the true nature of individuals' motivational orientations toward behaviors. Individuals can therefore identify varying levels of autonomous and controlled reasons for acting, the relative contribution of which likely determines the extent to which individuals will persist with or desist from the behavior in the long run.

Self-determination theory suggests that fostering autonomous forms of motivation for behaviors through environmental supports that foster autonomous reasons will lead to effective self-regulation (Ng et al., 2012). Research has demonstrated that engaging in behavior for largely autonomous reasons is associated with uptake and persistence with health-related behavior in a number of behavioral domains (e.g. Chatzisarantis, Hagger, Biddle, Smith, & Wang, 2003; Hagger, Chatzisarantis, & Harris, 2006a; Hagger et al., 2012; Teixeira, Carraca, Markland, Silva, & Ryan, 2012; Williams et al., 2006). The environmental conditions that likely affect motivation to engage in different health behaviors may vary. Some behaviors may have a greater tendency to engender autonomous reasons for engaging in them while others may have a greater propensity to be determined by external forces (Ryan & Deci, 2000). While autonomous motivation may be important for successful engagement in, and persistence with, many behaviors, it may be that the relative contribution of the different forms of motivation varies. While some researchers have compared the effects of autonomous forms of motivation on more than one health behavior (Hagger et al., 2006a; Hagger, Chatzisarantis, & Harris, 2006b), they have tended to focus only on a handful of conceptually related health behaviors (e.g. behaviors like exercise and healthy eating that are related to energy balance). To date there has been no systematic research that has examined the relative contribution of autonomous and controlled forms of motivation to behavioral engagement for multiple health behaviors. This is important as it will provide an indication as to whether some behaviors tend to be acted upon for more autonomous rather than controlled reasons, and vice versa. In other words, are there behaviors that tend to engender a more autonomous regulation in terms of the typical motivational regulation adopted by individuals when acting relative to others that tend to foster a more controlled regulation? This is important theoretically because a general premise of self-determination theory is that the motivational processes are consistent across contexts and actions. It is also important for intervention design as it will provide evidence that means to support autonomy will generalize across behavioral domains. A primary aim of the current research, therefore, is to test the relative effects of autonomous and controlled forms of motivation on action in multiple health domains.

### 1.3. Causality orientations and individual differences

Another important premise of self-determination theory is individuals' behavior is also determined by generalized and stable motivational orientations (Deci & Ryan, 1985a; Thrash & Elliot, 2002). The theory proposes that individuals differ in the extent to which they generally interpret behaviors as autonomy- or control-oriented. These are generalized, trait-like perceptions regarding the perceived cause or origin of behavior. A good example of these traits is *causality orientations*, which is tapped by the general causality orientations scale (Deci & Ryan, 1985a). A self-determined or autonomy orientation reflects an individual's propensity to experience environmental contingencies and actions as autonomous and supporting psychological needs. In contrast, a control orientation reflects the tendency to interpret situations and behaviors as controlled by external events and not able to satisfy, or even thwarting, psychological needs. Causality orientations are considered separate from the motivational orientations observed for individuals for specific behaviors; rather they are expected to contribute to, but are not necessarily deterministic of, the type of motivation experienced in a given context. In other words, the type of motivation, autonomous or controlled, is likely to be determined by contingencies in the environment in which the behavior is conducted and the features of the behavior itself as well as causality orientations (Deci & Ryan, 1985a).

The extent to which the dispositional and environmental factors will affect the impact that forms of motivation in a specific context have on behavior has not been formally investigated. Dispositional forms of autonomous and controlled motivation like causality orientations may serve to magnify or diminish the effects of motivation engendered by the situation or environment on action (Hagger & Chatzisarantis, 2011). In other words, dispositional motivation may serve to moderate the effects of situational autonomous or controlled forms of motivation on behavior, a fact that has been supported empirically. However, there are no studies that have systematically examined the relative contribution of generalized, trait-like autonomous or controlled orientations and situational autonomous motivation on behavior. In the present study we seek to fill this gap by examining whether we can identify groups of individuals with a general tendency to engage in their behavior for autonomous or controlled reasons, and are therefore more autonomy- or control-oriented. Furthermore, we plan to examine whether membership of the autonomy- or control-oriented groups moderates the relative contribution of autonomous or controlled motivational regulations on behavior across a number of health behaviors.

### 1.4. The present study

The purpose of the present study was to examine the effects of autonomous and control forms of motivation across multiple health behaviors. We were interested in establishing whether these effects were consistent across multiple behaviors, as predicted by self-determination theory, or whether the effects varied depending on behavior type. In addition, we also aimed to test whether individual differences in autonomy- and control-oriented motivation moderated the effects of behavioral regulations on behavior across multiple health-related behaviors. We adopted a process model for the effects of autonomous and controlled motives on behavior. The model is based on integrated models of motivation in health behavior in which intention is conceptualized as the most proximal predictor of behavior (Hagger, 2013; Hagger & Chatzisarantis, 2009b, 2014; Hoyt, Rhodes, Hausenblas, & Giacobbi, 2009; Jacobs, Hagger, Streukens, De Bourdeaudhuij, & Claes, 2011). Intention reflects the extent to which individuals will make plans and invest effort in engaging in a target behavior in future. Social-cognitive models of health behavior such as protection motivation theory (Rogers, 1975) and the theories of reasoned action (Fishbein & Ajzen, 2009) and planned behavior (Ajzen, 2011) typically include intention as a mediator of the effect of distal factors (e.g. beliefs, motivational factors, dispositions) on behavior. The inclusion of intention enables researchers to model the extent to which the distal factors are involved in future deliberation over self-regulated behavior; effects that are unmediated by intention possibly reflect processes that occur outside the more deliberative, reflective processes and more likely reflect more spontaneous, non-conscious and impulsive processes (Hagger et al., 2006a).

**Figure 1.**
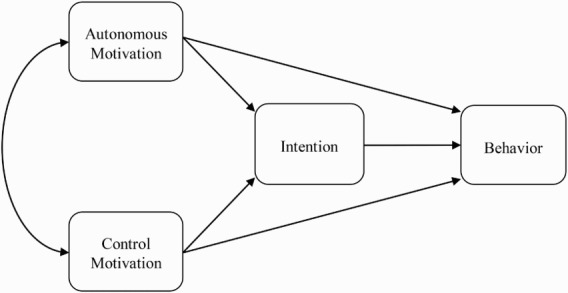
Process model of effects of autonomous and controlled forms of motivation on behavior mediated by intentions.

Note: Unidirectional arrows represent hypothesized paths. Bidirectional arrow represents a correlation.

In our proposed process model, autonomous and controlled forms of motivation reflect distal influences on health-related behaviors. Intention is proposed to mediate the effects of the two forms of motivation on behavior. To the extent that the direct effects of autonomous and controlled motivation on behavior are mediated by intention, we have sharp confirmation that autonomous and controlled forms of motivation affect behavior by influencing the deliberative processes preceding action. The process suggests that individuals' motivational orientations tend to bias their decision-making process so that their intentions match their motives. Individuals are, therefore, likely to bring their intentions to engage in future behavior into line with their motivational orientations. Research has suggested that individuals can and do make the distinction between autonomous and controlled motives at the beliefs level in health behavior (McLachlan & Hagger, 2010, 2011). The fundamental driving force behind the link between motivation and action is satisfaction of psychological needs (Hagger et al., 2006a). Individuals that view actions as an opportunity to satisfy basic psychological needs will tend to form intentions to engage in those behaviors in the future. We expect a significant indirect effect of autonomous motivation on behavior mediated by intention. However, some behaviors may tend to be those that are likely reinforced by external contingencies or perceived pressures. As a consequence intentions may be formed on the basis of controlled motivational regulations. In such cases, a significant indirect effect of controlled motivational regulations on behavior is expected. Model hypotheses are summarized in Figure 1.

In the current research we will test the process model in multiple health-related behaviors (*N* = 20) relevant to important health outcomes (controlling calorie intake to control weight, eating low-fat foods, wearing a seat belt when a passenger in cars/taxis/mini-buses, getting a good night's sleep, keeping alcohol intake within daily guideline limits, wearing a condom when having sexual intercourse, washing hands before preparing or handling food, regularly going for a walk to relax and unwind, brushing teeth every day, avoid eating “junk” food, avoiding consumption of caffeine, using stairs instead of an elevator or escalator, washing hands after going to the toilet, taking dietary supplements to maintain a healthy diet, exercising regularly for more than 30 minutes at a time, planning work in advance to reduce stress, sitting with correct posture to avoid back pain, avoiding foods high in sodium/salt, eating foods containing sufficient dietary fiber, five portions of fruit and vegetables per day). The behaviors have been identified in previous research as key behaviors linked to salient adaptive health outcomes in undergraduate students, the target population of the current study (Finlay, Trafimow, & Villarreal, 2002; Hagger, Anderson, Kyriakaki, & Darkings, 2007; Trafimow & Finlay, 1996). Given that the majority of research on self-determination theory in health contexts suggests that autonomous forms of motivation is linked to behavioral persistence, we expect that the path from autonomous motivation to behavior mediated by intention will be the most pervasive and consistent across the health behaviors with a lesser role for the influence of controlled forms of motivation, again mediated by intentions. We expect the proposed pattern of effects to be relatively consistent across health behaviors. Furthermore, in the majority of cases we expect intention to completely mediate the effects of forms of motivation from self-determination theory on behavior because many require considerable long-term planning and are not necessarily sensitive to automatic or habitual action. However, some behaviors may be more likely controlled by automatic processes. For example, Keatley, Clarke, and Hagger (2012, 2013) found that the effect of implicit autonomous motives was more pervasive for behaviors like wearing a seat belt when a passenger in vehicles and brushing teeth every day, probably because these behaviors tend to be highly dependent on habitual action. It may be that there are significant direct effects unmediated by intentions for these types of behavior. The adoption of the process model, in which we estimate direct and indirect effects of motivation types on behavior across multiple domains, provides us with the opportunity to test which of the effects are and therefore which process is the most relevant to explain particular behaviors.

In addition to examining the relative contribution of autonomous and control regulations to health behavior across multiple health behaviors, we also plan to examine whether the tendency to base one's intentions on autonomous or controlled motives across multiple behaviors moderates the overall pattern of effects of the motivational regulations on behavior in the process model. We will adopt a novel within-participants correlational approach to investigate this question. Specifically, we will conduct a within-participants regression analysis for each individual to test the relative contribution of autonomous and control motivational regulations on intentions and behavior across all 20 behaviors. In the analysis the multiple behaviors act as the unit of analysis rather than the participant, as is the case in traditional between-participants analyses. Although the majority of participants likely based their intentions on autonomous motivation, we anticipate that a proportion of the sample based their intentions on controlled motivation. After segregating the sample into two groups on the basis of whether their intentions are largely based on autonomous or controlled forms of motivation, we will test the process model in each group and separately for each behavior. It is expected that these analyses will evaluate whether the basis of the intention influences the relative contribution that autonomous and controlled forms of motivation make to the prediction of health-related behavior. The study is expected to contribute to knowledge by providing a basis for determining, in general, whether the characteristics of the behavior or the characteristics of the individual are most pervasive in determining the extent to which health-related behaviors are predicted by autonomous or controlled forms of motivational regulation.

In summary, we hypothesized that autonomous motivational regulations will have consistent and pervasive effects on intentions and behavior across multiple health-related behaviors, with substantially smaller effects for control motivational regulation, consistent with the premises of self-determination theory. We also hypothesized that the effects of autonomous and control motivational regulation on intentions and behavior would vary according to participants' individual differences in autonomy and control motivational orientation, determined by within-participants analyses. Specifically, autonomy-oriented participants were expected to have substantially stronger effects of autonomous motivation on intentions and behavior across the different health behaviors relative to control-oriented participants. Analogously, control-oriented individuals are expected to have stronger effects of control motivational regulation on intentions and behavior relative to autonomy-oriented individuals.

## 2. Method

### 2.1. Participants and procedure

Undergraduate students (*N* = 175; males = 65; females = 109; mean age = 21.45, SD = 4.49) from two universities (University of Bedfordshire, UK and University of Queensland, Australia) agreed to participate in the study. In the first wave of data collection, participants were presented with an online questionnaire containing standardized measures of the motivational regulation constructs from perceived locus of causality and items measuring behavioral intention for 20 health-related behaviors salient to the student sample. In order to reduce common method variance and response fatigue, the online questionnaire was designed to present measures to participants in a random order by behavior and to give participants an enforced 60-second break every five minutes. Participants completed an individual difference measure of self-determined motivation at the end of the online questionnaire after completing the motivational regulation measures. Participants completed the measures in quiet laboratory conditions in groups of 30–40. Participants were followed-up in a second wave of data collection four weeks later by email. The email contained a brief introduction letter and a link to the online second-wave questionnaire. Participants were asked to click on the link and complete the questionnaire by providing an anonymous code comprising their date of birth, gender, and mother's maiden name that they specified during the first wave of data collection. Participants completed two-item self-report measures for each behavior. Ethical clearance for the study protocol was secured from the Institutional Review Boards of the participating universities.

### 2.2. Measures

#### 2.2.1. Perceived locus of causality

^1^We adapted Ryan and Connell's (1989) measure of perceived locus of causality in educational contexts to measure forms of autonomous and controlled motivation for each of the 20 health-related behaviors.^2^ Participants were presented with initial instructions: “Thank you for agreeing to participate in our survey on your opinions about your participation in everyday pass-times and behaviors. Everyone feels differently about this so there are no right or wrong answers, we are interested in your opinions.” They were next presented with a common stem: “I control my calorie intake to control my weight because … ” followed by eight reasons, two for each regulation style: external regulation (e.g. “ … I feel under pressure to control my calorie intake to control my weight”), introjected regulation (e.g. “ … I will feel guilty if I do not control my calorie intake to control my weight”), identified regulation (e.g. “ … I value the benefits of controlling my calorie intake to control my weight”), and intrinsic motivation (e.g. “ … I enjoy controlling my calorie intake to control my weight”). Responses were measured on four-point scales ranging from “not true at all” (1) to “very true” (4). The average Spearman–Brown reliability (*ρ*) estimates for these items across all behaviors were indicative of adequate reliability (intrinsic motivation, Mdn *ρ* = .777; identified regulation, Mdn *ρ* = .890; introjected regulation, Mdn *ρ* = .824; external regulation, Mdn *ρ* = .766). For the main analyses, we reduced the perceived locus of causality scales to two autonomous and controlled motivational regulation indices using weighted composites (e.g. Guay, Mageau, & Vallerand, 2003; Hagger et al., 2006a). The average of the two perceived locus of causality items was computed for each scale. The autonomous motivational regulation index was computed as the sum of the intrinsic motivation scale weighted by a factor of two and the identified regulation item. Similarly, the control motivational regulation index was computed as the sum of the external regulation scale weighted by two and the introjected regulation scale.

#### 2.2.2. Intention

Behavioral intentions were measured on two items: “I intend to control my calorie intake to control my weight in the next 4 weeks” and “I plan to control my calorie intake to control my weight in the next 4 weeks” using six-point scales anchored by 1 (“unlikely”) and 6 (“likely”). The average reliability estimate of this scale across all behaviors was satisfactory (Mdn *ρ* = .965).

#### 2.2.3. Behavior

Four weeks later, participants self-reported their behavior for each of the 20 behaviors on a single item (e.g. “In the course of the past four weeks, how often have you controlled your calorie intake to control your weight”) using seven-point scales with scale points “almost every day”, “most days”, “on about half the days”, “a few times, but less than half the days”, “a few times”, “once or twice”, and “never”.

#### 2.2.4. Dispositional motivational orientations

Individual differences in self-determined motivation were assessed using the choice subscale of the Self-Determination Scale (Thrash & Elliot, 2002). Participants were presented with a series of five pairs of statements, labeled “A” and “B” (e.g. “A. I always feel like I choose the things I do. B. I sometimes feel that it's not really me choosing the things I do”). For each pair, participants were required to indicate the extent to which they agreed with one of the statements on a five-point scale 1 (“only ‘A’ feels true”) and 5 (“only ‘B’ feels true”) endpoints. Items were coded such that higher scores represented greater self-determination. The alpha reliability estimate for this scale was satisfactory (*α* = .813).

## 3. Results

### 3.1. Preliminary analyses

Attrition across the initial and four-week follow-up waves of data collection due to absence was 24.29% leaving a final sample of 140 (males = 53; females = 87; mean age = 21.08, SD = 3.94). Analyses for attrition bias revealed no significant differences in the proportion of gender (*χ* ^2^ (174) = .077, *p* = .782) or age (*t*(172) = 4.001, *p* = .097) across the waves.

### 3.2. Main analyses

#### 3.2.1. Between-participants tests of the process model

Between-participants zero-order correlations among the study variables are presented in Table 1. A series of between-participants path analyses were conducted to test the hypotheses of the proposed process model (see Figure 1). The analysis was conducted using manifest averaged variables and the proposed model specified a priori and applied to the data. The model was estimated using the EQS v. 6.1 structural equation modeling software. Results are presented in Table 2. The theory variables accounted for approximately half of the variance in intentions across all the behaviors (*M R* ^2^ = .499, *p* < .001).^3^ Autonomous motivational regulation had the strongest effect on intentions (*M β* = .573, *p* < .001) while controlled motivational regulation had a much smaller effect (*M β* = .158, *p* < .001) across all the behaviors. Intentions statistically significantly predicted behavior (*M β* = .285, *p* < .001). There were statistically significant indirect effects of autonomous (*M β* = .203, *p* < .001) and controlled (*M β* = .061, *p* < .001) motivational regulations on behavior, although the effect for autonomous motivation was larger. There was a small but statistically significant direct effect of autonomous motivational regulation on behavior (*M β* = .131, *p* < .001) but no statistically significant effect for controlled motivation. The statistically significant direct and indirect effects for autonomous motivational regulation on behavior suggest partial mediation for this variable across all behaviors. Overall, the averaged effect of autonomous motivational regulation was substantial as a predictor of intention and behavior, with the effect of autonomous motivational regulation on behavior partially mediated by intention. Controlled motivational regulation also had statistically significant effects on intentions and behavior mediated by intention, but the overall impact was far smaller than that for autonomous motivation, and, for many behaviors, the effect was not significant.

**Table 1.** Zero-order between- and within-participants correlations and standard errors among the self-determination theory constructs, intention, and behavior.

Factor	1	2	3	4
1. Autonomous motivation	−	.503** (.033)	.652** (.049)	.320** (.038)
2. Control motivation	.513** (.019)	−	.451** (.042)	.162** (.043)
3. Intention	.731** (.012)	.668** (.017)	−	.403** (.035)
4. Behavior	.510** (.016)	.483** (.017)	.660** (.014)	−

Notes: Mean between-participant correlations among the self-determination theory components, intention, and behavior are shown above the principal diagonal; Median within-participant correlations among the self-determination theory components, intention, and behavior are shown below the principal diagonal. Values in parentheses are standard errors.

*p < .05.

**p < .01.

**Table 2.** Standardized path coefficients (*β*) and *R* ^2^ values from full-sample between-participants path analyses predicting intentions and behavior using the self-determination theory motivational constructs for each behavior.

Behavior	Direct effects					Indirect effects		*R*^2^	
	*β*_AUT→INT_	*β*_CON→INT_	*β*_AUT→BEH_	*β*_CON→BEH_	*Β*_INT→BEH_	*β*_AUT→INT→BEH_	*β*_CON→INT→BEH_	INT	BEH
Calorie	.641***	.294***	.233*	.184*	.385***	.246***	.113**	.693***	.512***
Low-fat diet	.668***	.231***	.102	−.076	.503***	.336***	.116	.612***	.305***
Seat belt	.100	.150	.045	.000	.139	.014	.021	.050	.024
Sleep	.507***	−.129	−.015	−.130	−.294**	.149**	−.038	.223***	.095
Alcohol	.652***	.136*	.292**	−.311***	.305**	.199**	.042	.539***	.215**
Condoms	.298***	.521***	−.122	.065	.652***	.194***	.340***	.522***	.403***
Handwash – food	.400***	.209*	.051	.188*	.410***	.164***	.086*	.282***	.297***
Walks	.808***	.086	−.147	.066	.435**	.351**	.037	.712***	.123**
Toothbrushing	.305***	.105	−.025	−.110	.143	.044	.015	.121***	.026
Junk food	.502***	.262***	.233*	−.102	.335***	.168**	.088*	.460***	.215***
Caffeine	.801***	.044	.214	.180	−.343***	.171	−.009	.604***	.126**
Stairs	.752***	.143**	−.067	−.128	.538***	.405***	.077*	.684***	.192***
Handwash – toilet	.115	.225*	−.017	.245**	.193*	.022	.043	.088	.199**
Supplements	.795***	.084	.365**	−.130	.408*	.325**	.034	.739***	.429***
Exercise	.456***	.117	.184*	−.074	.387***	.176***	.045	.240***	.241***
Plan work	.733***	.102	.470***	−.318***	.110	.080	.011	.634***	.205***
Posture	.705***	.179**	.185	−.205	.382**	.269*	.068	.722***	.170*
Sodium/salt	.738***	.158**	.138	−.174	.421**	.311**	.067	.732***	.194***
Food fiber	.750***	.125*	.313*	−.110	.183	.138	.023	.668***	.184**
Fruit and veg	.728***	.122*	.196	−.041	.402***	.293**	.049	.647***	.301***
*M* values	.573***	.158***	.131***	−.049	.285***	.203***	.061***	.499***	.222***

Notes: AUT = Autonomous motivation; CON = Control motivation; INT = Intention; BEH = Behavior; Calorie = controlling calorie intake to control weight; Low-fat diet = eating low-fat foods; Seat belt = wearing a seat belt when a passenger in cars/taxis/mini-buses; Sleep = Getting a good night's sleep (more than seven hours); Alcohol = Keeping alcohol intake within daily guideline limits; Condoms = Wearing a condom when having sexual intercourse; Handwash – food = Washing hands before preparing or handling food; Walk = Regularly going for a walk to relax and unwind; Toothbrushing = Brushing teeth every day; Junk food = Avoid eating ‘junk’ food; Caffeine = Avoiding consumption of caffeine; Stairs = Using stairs instead of an elevator or escalator; Handwash – toilet = Washing hands after going to the toilet; Supplements = Taking dietary supplements to maintain a healthy diet; Exercise = Exercising regularly for more than 30 minutes at a time; Plan work = Planning work in advance to reduce stress; Posture = Sitting with correct posture to avoid back pain; Sodium/salt = Avoiding foods high in sodium/salt = Eating foods containing sufficient dietary fiber; Fruit and veg = Eat five portions of fruit and vegetables per day.

*p < .05.

**p < .01.

***p < .001.

There were some notable effects for individual behaviors that deviated from the overall trend. Autonomous motivational regulation had no impact on intention or behavior for two behaviors: seat-belt use and washing hands after going to the toilet. In fact, for seat-belt use, none of the motivational orientations predicted intentions or behavior, and the intention–behavior relationship was non-significant. Similarly, the intention–behavior relationship for brushing teeth every day, planning work in advance to reduce stress, and eating foods with sufficient fiber behaviors was not statistically significant. Wearing a condom when having sexual intercourse and washing hands after going to the toilet were the only behaviors where the effect of controlled motivational regulation was stronger than that of autonomous motivational regulation. Other than these exceptions, the pattern of effects across the behaviors largely conformed to expectations with a stronger effect of autonomous motivational regulation relative to control motivational regulation, and a significant intention–behavior relationship.

#### 3.2.2. Within-participants analyses

In addition to the between-participants regressions analyses to test the process model, the present data set also permitted a test of the model for each individual participant across the 20 behaviors using within-participants analyses (Finlay et al., 2002; Hagger & Chatzisarantis, 2006; Sheeran, Trafimow, Finlay, & Norman, 2002; Trafimow & Finlay, 1996).^4^ Zero-order within-participants correlations among the motivational orientations, intention, and behavior variables are depicted in Table 1. As with the between-participants correlations, within-participants correlations among the study constructs were all positive and significant. These correlations were used as input matrices for a within-participants regression analysis examining the relative contribution of the autonomous and controlled motivational orientations on intention and behavior. Separate analyses were conducted for intentions and behavior. Consistent with the path analyses for the process model, the regression analysis with intention as the dependent variable included autonomous and controlled motivational regulations as the independent variables. The analysis for behavior included intentions alongside the two motivational regulations as predictors.^5^

Results from the within-participants regression analyses for intention indicated that a substantial proportion of the variance in intention was explained across each participant (Mdn *R* ^2^ = .534, *p* < .001). Autonomous (Mdn *β* = .515, *p* < .001) and controlled (Mdn *β* = .209, *p* < .001) motivational regulations contributed significantly to the prediction of intention. Turning to the regression with behavior as the dependent variable, intention (Mdn *β* = .588, *p* < .001) was the only significant predictor of behavior and explained a significant proportion of the variance in behavior in each analysis (Mdn *R* ^2^ = .509, *p* < .001). Autonomous (Mdn *β* = .079, *p* > .05) and control (Mdn *β* = −0.011, *p* > .05) motivational regulations had negligible effects on behavior. These results were similar to those found in between-participants tests of the hypothesized process model. The only exception was the lack of a direct effect of autonomous motivational orientation on behavior.

#### 3.2.3. Testing study hypotheses for autonomy- and control-oriented participants

In order to test the hypothesis that the majority of participants would primarily base their intentions on autonomous motivational regulation and a minority would form intentions on control motivational regulation, we segregated the sample on the basis of the within-participants regression analyses. Participants for whom the within-participants correlation between autonomous motivational regulation and intention was stronger than the within-participants correlation between control motivational regulation and intention were classified as autonomy-oriented. Similarly, participants for whom the within-participants correlation between control motivational regulation and intention was greater than their within-participants autonomous motivational orientation–intention correlation were classified as control-oriented. We then conducted separate between-participants path analyses of the process model for each behavior in each segregated sample. For each behavior, therefore, two analyses were conducted; one for the autonomy-oriented participants and one for the control-oriented participants. The segregation procedure revealed that the majority of the sample (*n* = 86, 61.40%) were classified as autonomy-oriented, while a substantial minority (*n* = 54, 38.60%) were classified as control-oriented. The choice subscale of the Self-Determination Scale provided some external validation of the classification. Participants classified as autonomy-oriented reported higher scores on the choice questionnaire (*M* = 2.352, SD = .735) relative to those classified as control-oriented (*M* = 2.141, SD = .778), *t*(138) = 1.61, *p* = .110.

We conducted the between-participants path analyses of the process model using EQS ver. 6.1 again for each of the behaviors and in each separate subsample. Results of the path analyses for each subsample are given in Table 3. Summarizing the overall results, autonomous motivational regulation had a statistically significant influence on intentions in all but one of the behaviors in the autonomy-oriented sample (Mdn *β* = .652, *p* < .001) and all but two of the behaviors in the control-oriented sample (Mdn *β* = .471, *p* < .001). There was no significant difference in the *β*-coefficient for the effect of autonomous motivational regulation on intentions across the groups (Mann–Whitney *U* = 152, *z* = −1.299, *p* = .201). However, the effect of control motivation on intention was statistically significant for 11 of the behaviors in the control-oriented sample (Mdn *β* = .235, *p* < .05) but only for six of the behaviors in the autonomy-oriented sample (Mdn *β* = .093, *p* < .001). There was also a statistically significant difference in the magnitude of the betas across the groups (Mann–Whitney *U* = 58, *z* = −3.842, *p* < .001). However, autonomous motivational orientation had the highest *β*-coefficient in the prediction of intention for the majority of behaviors in both autonomy-oriented (*n* = 16; 80.0%) and control-oriented (*n* = 13; 65.0%) samples, a difference that was not statistically significant (*χ* ^2^ = 1.129, *p* = .288). There were no other statistically significant differences in the average direct or indirect effects in the model. There were also no significant differences in the average proportion of variance explained in intentions and behavior in the models across the two groups. Overall, the results indicated little evidence for any moderation of the overall effects of autonomous motivational regulation on intention and behavior by the autonomy- and control-oriented samples. While there seemed to be a greater effect for control motivational regulation on intentions for a statistically significant proportion of the behaviors in the control-oriented sample, autonomous motivational regulation still made a significant contribution and was the dominant effect in most of the behaviors in both samples.

**Table 3.** Results of between-participants path analyses predicting intention and behavior for the autonomy-oriented and control-oriented participants based on within-participant regressions.

Behavior	Direct effects					Indirect effects		*R*^2^	
	*β*_AUT→INT_	*β*_CON→INT_	*β*_AUT→BEH_	*β*_CON→BEH_	*Β*_INT→BEH_	*β*_AUT→INT→BEH_	*β*_CON→INT→BEH_	INT	BEH
Calorie	.376***	.420***	−.096	.151	.522***	.196**	.219**	.518***	.326***
	.538***	.427***	.210	.481**	.002	.001	.001	.764***	.407***
Low-fat diet	.702***	.**127**	.063	−.026	.583***	.409***	.074	.578***	.386***
	.493***	.474***	.159	−.047	.295	.146	.140	.717***	.156
Seat belt	.121	.144	.018	.009	.201	.024	.029	.055	.043
	.046	.170	.090	−.020	.054	.002	.009	.041	.010
Sleep	.483***	−.233*	−.025	−.131	.316**	.153*	−.074	.203***	.118*
	.409***	.166	−.018	−.072	.217*	.089	.036	.263***	.038
Alcohol	.713***	.042	.463**	−.317**	.139	.099	.006	.539***	.254**
	.398**	.433***	−.026	−.335*	.611**	.243***	.265*	.584***	.186**
Condoms	.376***	.420***	−.096	.151	.522***	.196**	.219**	.518***	.326***
	.271***	.590***	−.039	−.199	.897***	.243**	.530**	.551***	.567***
Handwash – food	.468***	.**046**	.079	.183*	.410***	.181***	.018	.245***	.278***
	.192**	.565***	.027	.190*	.405***	.078*	.229***	.449***	.314***
Walks	.647***	.098	−.110	.103	.382**	.248**	.038	.445***	.125**
	.800***	.178**	−.096	−.097	.536**	.429**	.095	.852***	.153**
Toothbrushing	.318***	.052	−.082	−.088	.247*	.079	.013	.113*	.060
	.206	.158	.299*	−.377**	.003	.001	.001	.096	.132**
Junk food	.630***	.094	.192	−.030	.398**	.251**	.037	.470***	.284***
	.**223**	.568***	.175	−.121	.235	.052	.134	.513***	.082
Caffeine	.785***	−.080	.176	−.298**	.206	.162	−.016	.565***	.163
	.815***	.030	.284	−.558**	.270	.220	.008	.704***	.137
Stairs	.658***	.143	−.141	−.066	.404**	.266**	.058	.520***	.093***
	.755***	.183*	−.141	−.047	.607**	.458**	.111	.797***	.208***
Handwash – toilet	.115	.168	−.045	.283**	.243*	.028	.041	.063	.154**
	.074	.356**	.131	.057	.130	.010	.046	.156**	.058
Supplements	.886***	.024	.488**	−.215*	.437**	.387**	.010	.817***	.538***
	.653***	.178	.150	.062	.306	.200	.055	.635***	.232***
Exercise	.447***	.152	.196	−.068	.317**	.142*	.048	.260***	.185**
	.450***	.082	.167	−.044	.524***	.236*	.043	.211***	.378**
Plan work	.816***	−.001	.411*	−.367**	.150	.122	.000	.666***	.213**
	.546***	.286*	.441*	−.211	.063	.034	.018	.600***	.135
Posture	.814***	.034	−.042	−.017	.601**	.489**	.021	.708***	.308**
	.738***	.158**	.138	−.174	.421**	.311**	.067	.732***	.194***
Sodium/salt	.730***	.166*	.185	−.250*	.389*	.284*	.065	.706***	.194*
	.765***	.127	−.105	.069	.503*	.385*	.064	.765***	.217*
Food fiber	.793***	.**052**	.429*	−.160	−.024	−.019	−.001	.666***	.138
	.449***	.485***	.198	−.105	.434*	.195	.210	.738***	.272*
Fruit and veg	.762***	.092*	.104	−.080	.522***	.397**	.048	.655***	.332***
	.525***	.334**	.275	.153	.139	.073	.046	.662***	.276**
*M* values	.652***	.093*	.071*	−.067	.385***	.189**	.033	.519***	.204***
	.472***	.235***	.144*	−.059	.301***	.171**	.060	.618***	.190***

Notes: For each behavior, statistics given on the top line are for the autonomy-oriented sample and the bottom line for the control-oriented sample; statistics given are the median average; bold values represent statistically significant differences in coefficients across the autonomy- and control-oriented samples (*p* < .05). AUT = Autonomous motivation; CON = Control motivation; INT = Intention; BEH = Behavior; Calorie = controlling calorie intake to control weight; Low-fat diet = eating low-fat foods; Seat belt = wearing a seat belt when a passenger in cars/taxis/mini-buses; Sleep = Getting a good night's sleep (more than seven hours); Alcohol = Keeping alcohol intake within daily guideline limits; Condoms = Wearing a condom when having sexual intercourse; Handwash – food = Washing hands before preparing or handling food; Walk = Regularly going for a walk to relax and unwind; Toothbrushing = Brushing teeth every day; Junk food = Avoid eating ‘junk’ food; Caffeine = Avoiding consumption of caffeine; Stairs = Using stairs instead of an elevator or escalator; Handwash – toilet = Washing hands after going to the toilet; Supplements = Taking dietary supplements to maintain a healthy diet; Exercise = Exercising regularly for more than 30 minutes at a time; Plan work = Planning work in advance to reduce stress; Posture = Sitting with correct posture to avoid back pain; Sodium/salt = Avoiding foods high in sodium/salt = Eating foods containing sufficient dietary fiber; Fruit and veg = Eat five portions of fruit and vegetables per day.

*p < .05.

**p < .01.

***p < .001.

Finally, we tested for differences in the magnitude of the path coefficients in the process model for each sample across the autonomy- and control-oriented samples for each behavior. In all cases, 95% confidence intervals for the coefficients were used with a lack of overlap in the upper and lower bounds indicative of a statistically significant difference. We only found four differences across the entire sample (see Table 3). The effect of control motivational orientations on intentions was statistically significantly higher in the control-oriented sample for eating a low-fat diet, washing hands before handling food, and eating foods with sufficient fiber behaviors. In addition, the effect of autonomous motivation on intention was statistically significantly higher in the autonomy-oriented sample for eating junk food only. There were no other statistically significant differences, suggesting that effects at the individual level were largely consistent with trends observed in the analysis of the averaged effects across the behaviors for each sample.

## 4. Discussion

The purpose of the present study was to test the relative contributions of autonomous and controlled motivational regulations on behaviors in multiple health domains. We also wanted to test whether these effects varied across behaviors according to participants whose intentions were based on autonomous motivational regulations and participants whose intentions were based on control motivational regulations in within-participants analyses. We hypothesized a process model, based on integrated models of health behavior (Hagger, 2013; Hagger & Chatzisarantis, 2009b, 2014), in which autonomous and control motivational regulations predicted behavior mediated by intentions. The study is unique as it is the first to systematically examine the relative contribution of autonomous and controlled motivational regulations in multiple health-related behaviors rather than an isolated behavior or small collections of behaviors. It is also the first to adopt both between- and within-participants approaches to examining the consistency of the effects across behaviors and across individuals. Finally, it is the first study to utilize a within-participants approach (Finlay et al., 2002; Hagger & Chatzisarantis, 2006; Sheeran et al., 2002; Trafimow & Finlay, 1996) to identify groups of individuals who are either autonomy- or control-oriented and observe the effects of the two groups on the relative contribution of each regulation type in individual behaviors.

Results support previous research in that autonomous motivation had the most pervasive effect on intentions and behavior across the majority of the health-related behaviors (Ng et al., 2012). There were some noteworthy idiosyncratic deviations from this trend for seat-belt use, wearing a condom when having sexual intercourse, washing hands after going to the toilet, eating foods containing sufficient dietary fiber, and planning work in advance to reduce stress. Within-participants regression analyses using the behavior as the unit of analysis also indicated the pervasive effect of autonomous motivation on intentions and behavior across the health for most participants. Segregating the sample on the basis of the relative contribution of autonomous and controlled motivational regulations on intentions and recalculating the between-participants path analyses for each behavior in each group revealed few deviations from trends found in the full sample. There was a significant difference in the effect for control motivational regulation on intention between the autonomy- and control-oriented samples, but this difference was of little consequence given that the direct and indirect effects of control motivational orientation on intention and behavior in each analysis were not significant. We also found few differences in individual coefficients from the process model across the two groups within each behavior, which supported the lack of findings in the analysis of the averaged coefficients. Overall, the pervasive effect of autonomous motivation on intentions and behavior was replicated in each analysis, consistent with the analyses for the full sample.

Present findings provide pertinent information on the processes by which autonomous and controlled forms of motivational regulations affect behavior across a number of health domains. Results are largely consistent with previous research examining the effects of motivational constructs from self-determination theory on health-related behaviors (Chatzisarantis *et al*., 2003; Hagger et al., 2006a, 2012; Teixeira, Carraca, *et al*., 2012; Williams et al., 2006). Specifically, the consistent effect for autonomous motivational regulations is supported in the current research, with lesser role for control-oriented motivation. This is consistent with the tenets of self-determination theory; autonomous forms of motivation tend to be related to behavioral engagement and persistence, the mechanism for which has purported to be that individuals will tend to take up and adhere to behaviors if their engagement in the behavior is perceived to be for autonomous reasons and, therefore, consistent with psychological needs (Deci & Ryan, 2000). Controlled motivational regulations are less likely to be behaviorally adaptive because the behavior tends to be perceived as inconsistent with psychological needs and may even thwart them. Our current research therefore supports the general tenets of self-determination theory and the importance of autonomous forms of motivation to health behavior in general (Ng et al., 2012).

Our research takes this premise a step further by examining the role of autonomous and control forms of motivational regulation in the context of a process model in which intentions mediate the effects of the two forms of motivational regulation on health behaviors. Our findings demonstrate that intentions are, generally, a significant predictor of health behavior and tend to mediate the effect of motivational regulations on actual behavior. The effect of control motivational regulation on intention, and on behavior mediated by intention, is substantially weaker. These findings have the potential to inform the further development of theory by demonstrating that individuals tend to bias their intention to engage in a health behavior in the future according to their motivational orientations toward the behavior (Hagger & Chatzisarantis, 2009b; Hagger, Chatzisarantis, et al., 2009). In other words, if people report autonomous motivational regulations with respect to a particular behavior, they will likely view it as satisfying psychological needs and bring their cognitions into line with their motivation. In this case, across a raft of health-related behaviors, individuals with autonomous motivational regulations are more likely to form intentions to engage in those behaviors in future. Those intentions are also related to future behavioral engagement and are, therefore, implicated in the process by which autonomous forms of motivation affect behavior.

An important implication of the current research is that it lends support for the generality of the proposed effects from self-determination theory, and the process model, across multiple behaviors (Ng et al., 2012; Williams et al., 2002). Many social-cognitive and motivational theories propose that their hypothesized effects and processes are ubiquitous and hold for multiple behaviors (Hagger & Chatzisarantis, 2009a). This is consistent with social-cognitive and information-processing metaphors for human motivation and decision-making, given appropriate information on the environmental stimuli and psychological antecedents of action proposed in the theory or model, and peoples' behavior should be eminently predictable, within the boundary conditions of the theory and in the absence of measurement error. Current results are consistent with these premises and suggest that, generally speaking, some idiosyncratic findings excepted, the hypotheses of self-determination theory and the process model are applicable to the multiple health-related behaviors. This is also consistent with previous research that has demonstrated consistency in the hypothesized effects of self-determination theory and the process model across some behaviors and groups (Chirkov & Ryan, 2001; Hagger, Chatzisarantis, Barkoukis, Wang, & Baranowski, 2005; Hagger, Chatzisarantis, et al., 2009).

The relative consistency in the effects across the current set of health-related behaviors notwithstanding, we identified some idiosyncratic variations in the effects for certain behaviors in the path analyses in the full sample for role of autonomous and controlled motivational regulations and intentions. Specifically, autonomous motivational regulations did not predict intentions or behavior for the seat-belt use and washing hands after going to the toilet behaviors. For these behaviors, neither motivational regulation had a pervasive effect on behavior, with weak effects for intentions. Similarly, the brushing teeth every day, planning work in advance to reduce stress, and eating foods containing sufficient dietary fiber behaviors were not predicted by intentions. To speculate, the small effect for intention may indicate that deliberative constructs such as motivation and intention are less relevant to these kinds of behaviors. It might be that some of these behaviors are, perhaps, more likely to be subject to non-intentional, automatic processes. This has been noted in recent analyses that have shown implicitly measured constructs such as attitudes and motivation to have a pervasive effect on intentions, but these effects tend to vary across behaviors (Keatley et al., 2012, 2013). This has led to researchers proposing dual-systems theories and models of behavior that account for both deliberative, reflective processes and spontaneous, impulsive processes (Hagger, 2013; Perugini, 2005; Strack & Deutsch, 2004). Such approaches provide potential to evaluate the extent to which behaviors may be controlled by motivational regulations and intentions, from the more deliberative perspective, or more non-conscious, impulsive influences. What is, therefore, important is the extent to which behaviors are controlled by these different processes and behaviors may vary in the relative contribution of each. We must, however, stress that current evidence points to remarkable consistency in the effects of the factors identified in self-determination theory and the proposed effects in the process model across behaviors, despite the small number of variations identified.

An important and unique contribution of the current research is the adoption of within-participants analyses to establish the extent to which the effects of the motivational regulations from self-determination theory and the process model are consistent across the 20 health behaviors for each individual. In fact, we utilized this analysis to demonstrate whether individuals differed in the relative contribution that autonomous and controlled forms of motivational regulation made to the behaviors and whether those individual differences would bias the pattern of effects for the motivational orientations in each behavior. Our within-participants analyses largely corroborated our findings for the between-participants analysis of the process model. Averaged effects revealed pervasive effects for autonomous motivational regulations on intentions and behavior, with a lesser role for control motivational regulations. Furthermore, even though we identified a substantial proportion of the sample as having a stronger effect for controlled motivational regulation on intentions across the behaviors, relative to the effect for autonomous motivational regulation, segregating the sample on that basis did not reveal any substantive differences in the pattern of effects in the process model in between-participants path analyses. These findings indicate that even among individuals who tended to base their intentions on control motivational regulation, the effect of autonomous motivational regulation remains substantial and pervasive. Furthermore, autonomous motivational regulation appears to “win out” in the prediction of behavior regardless of the groups into which individuals are classified based on their within-participants correlations. The current research does not provide strong evidence to support the proposal that individual differences in autonomy- and control-orientations, determined on the basis of within-participants correlations, will affect the extent to which behaviors are influenced by the different types of motivational regulation from self-determination theory. Results seem to be more consistent with the generalizability hypothesis; autonomous motivational regulation tends to have strong effects on action across health behaviors in groups of people and within individuals.

### 4.1. Strengths, limitations, and future research

The current research has a number of strengths including the adoption of an appropriate process model based on theory, tests of the model across multiple health-related behaviors, and the adoption of unique between- and within-participants analytic design. However, it would be remiss not to mention a number of limitations which need to be considered when evaluating the overall contribution of the research and the extent to which findings can be generalized. First, even though we used a unique and rigorous analytic means to segregate the sample into autonomy- and control-oriented participants based on within-participants regressions, an analysis that has been applied elsewhere (Finlay et al., 2002; Hagger & Chatzisarantis, 2006; Sheeran et al., 2002; Trafimow & Finlay, 1996), the classification was not corroborated by scores on the choice subscale of the Self-Determination Scale (Thrash & Elliot, 2002). Specifically, although there were differences in the levels of dispositional choice across the two groups the analysis did not reach significance and the effect size was relatively small. We cannot, therefore, claim definitively that the segregation led to sufficient differentiation of the sample based on dispositional autonomy- and control-orientations. However, we must also acknowledge that choice alone does not sufficiently characterize an autonomous or controlled motivational disposition. A better scale to use would have been the causality orientations scale (Deci & Ryan, 1985a). We cannot, therefore, definitively say we have strong evidence that our analysis led to adequate classification into the two groups. We look to future research to provide adequate tests of concurrent validity for the classification of individuals into autonomy and control motivational orientations using within-participants analyses. This will serve to lend converging evidence for the findings in the current analysis.

Another limitation is the relatively short-term follow-up of the behavioral data. While four weeks is still sufficient to establish patterns of effect and has some interest for practitioners wishing to get people “on the road” to engaging in health-related behaviors, behavioral maintenance is important for individuals to glean long-term health outcomes. This problem is rife in tests of theories and models in health psychology and the multitude of “shortitudinal” studies has received criticism and calls for more longer-term follow-ups (Sniehotta, 2014). Another limitation is the correlational, prospective design which means that the causal and deterministic nature of the results should not be inferred. Future research that adopts intervention and experimental designs, such as manipulating autonomy support to promote autonomous motivation (e.g. Chatzisarantis & Hagger, 2009; Teixeira, Silva, Mata, Palmeira, & Markland, 2012), would provide useful confirmatory data to support the direction of effects proposed by self-determination theory and the process model tested here. Finally, responses of the current sample to the study measures may have been subject to measurement error due to using common methods to tap the study constructs and response fatigue (Hagger & Chatzisarantis, 2009a). The randomized presentation of items and the provision of enforced rest periods during the course of the study were designed to allay these concerns, but we have to identify this as a possible limitation of the study and had the potential to increase the error variance in the tested effects.

## Notes

1. All measures are provided in the full questionnaire as supplemental materials (see appendix).

2. The example items provided make reference to eating a low-calorie diet as the target behavior, details of all items and behavioral operationalization are provided in the full questionnaire as supplemental materials (see appendix).

3. The figures represent the mean average of the effects and *R* ^2^ values across the 20 behaviors.

4. A potential confound in within-participants analyses is that the target behavior used in the between-participants analyses was also used in the within-participants calculations. This means that the between-participants and within-participants analyses would include a ‘shared’ cell representing the common behavior. This potentially creates problems of independence in the analysis. Solutions to this problem include the adoption of a ‘jackknife’ procedure in the within-participants calculations that eliminated the shared cell and minimized overlap between the dependent variable in the between-participants analysis and the behaviors used in the classification of the sample from the within-participants analyses. Simulation and ‘real-world’ studies have demonstrated that the degree of dependence exhibited in studies using between- and within-participants analyses is promotional to sample size and number of items, in this case, behaviors, used in the study. A minimum of 15 participants and 15 behaviors was found to be sufficient in such analyses to ensure that the level of dependence remained relatively trivial. Given that the number of behaviors (*N* = 20) and participants (*N* = 140) we adopted in the current study exceeded the recommended number, we expected levels of dependence to be negligible.

5. Syntax files for the SPSS computer program used in the calculation of the determinants, cofactors, and inverse matrices for the within-participants analyses are available on request.

## Appendix

**PART 1: YOUR OPINIONS ABOUT YOUR EVERYDAY PASS TIMES AND BEHAVIOURS**

Thank you for agreeing to participate in our survey on your ***opinions*** about your participation in *everyday pass-times* and *behaviours*. Everyone feels differently about this so there are no right or wrong answers, we are interested in your ***opinions***. Do not spend too long on any one statement and give the response that best describes your feelings. All responses are strictly ***confidential***, and please answer ***all the questions***. For each pass time/behaviour please read all of the statements and **CIRCLE A NUMBER** for each.

**Controlling your calorie intake to control your weight**

(Remember to circle a number for **every** reason)

**Table:**

**I control my calorie intake to control my weight because … **	**Not true at all**			**Very true**
… I enjoy controlling my calorie intake to control my weight	**1**	**2**	**3**	**4**
… I value the benefits of controlling my calorie intake to control my weight	**1**	**2**	**3**	**4**
… I will feel guilty if I do not control my calorie intake to control my weight	**1**	**2**	**3**	**4**
… people I know well (e.g., friends, parents etc.) say I should control my calorie intake to control my weight	**1**	**2**	**3**	**4**
… it is fun to control my calorie intake to my control weight	**1**	**2**	**3**	**4**
… .I think it is important to make the effort to control my calorie intake to control my weight	**1**	**2**	**3**	**4**
… I will feel ashamed if I do not control my calorie intake to control my weight	**1**	**2**	**3**	**4**
… I feel under pressure to control my calorie intake to control my weight from people I know well (e.g., friends, parents etc.)	**1**	**2**	**3**	**4**

**Table:**

**Read the statements below and circle the number on the right that best describes your answer**	**Strongly Disagree**						**Strongly agree**
I intend to control my calorie intake to control my weight in the next 4 weeks	**1**	**2**	**3**	**4**	**5**	**6**	**7**
I plan to control my calorie intake to control my weight in the next 4 weeks.	**1**	**2**	**3**	**4**	**5**	**6**	**7**

**Eating low-fat foods**

**Table:**

**I eat low-fat foods because … **	**Not true at all**			**Very true**
… I enjoy eating low-fat foods	**1**	**2**	**3**	**4**
… I value the benefits of eating low-fat foods	**1**	**2**	**3**	**4**
… I will feel guilty if I do not eat low-fat foods	**1**	**2**	**3**	**4**
… people I know well (e.g., friends, parents etc.) say I should eat low-fat foods	**1**	**2**	**3**	**4**
… it is fun to eat low-fat foods	**1**	**2**	**3**	**4**
… .I think it is important to make the effort to eat low-fat foods	**1**	**2**	**3**	**4**
… I will feel ashamed if I do not eat low-fat foods	**1**	**2**	**3**	**4**
… I feel under pressure to eat low-fat foods from people I know well (e.g., friends, parents etc.)	**1**	**2**	**3**	**4**

**Table:**

**Read the statements below and circle the number on the right that best describes your answer**	**Strongly Disagree**						**Strongly agree**
I intend to eat low-fat foods in the next 4 weeks	**1**	**2**	**3**	**4**	**5**	**6**	**7**
I plan to go eat low-fat foods in the next 4 weeks	**1**	**2**	**3**	**4**	**5**	**6**	**7**

**Wear a seat belt when a passenger in cars/taxis/mini-buses:**

**I wear a seat belt when in cars/taxis/mini-buses because … **	**Not true at all**			**Very true**
… I enjoy wearing a seat belt when a passenger in cars/taxis/mini-buses	**1**	**2**	**3**	**4**
… I value the benefits of wearing a seat belt when a passenger in cars/taxis/mini-buses	**1**	**2**	**3**	**4**
… I will feel guilty if I do not wear a seat belt when a passenger in cars/taxis/mini-buses	**1**	**2**	**3**	**4**
… people I know well (e.g., friends, parents etc.) say I should wear a seat belt when a passenger in cars/taxis/mini-buses	**1**	**2**	**3**	**4**
… it is fun to wear a seat belt when a passenger in cars/taxis/mini-buses	**1**	**2**	**3**	**4**
… .I think it is important to make the effort to wear a seat belt when a passenger in cars/taxis/mini-buses	**1**	**2**	**3**	**4**

**Table:**

	****Not true at all****			****Very true****
… I will feel ashamed if I do not wear a seat belt when a passenger in cars/taxis/mini-buses	**1**	**2**	**3**	**4**
… I feel under pressure to wear a seat belt when a passenger in cars/taxis/mini-buses from people I know well (e.g., friends, parents etc.)	**1**	**2**	**3**	**4**

**Table:**

**Read the statements below and circle the number on the right that best describes your answer**	**Strongly Disagree**						**Strongly agree**
I intend to wear a seat belt when a passenger in cars/taxis/mini-buses in the next 4 weeks	**1**	**2**	**3**	**4**	**5**	**6**	**7**
I plan to wear a seat belt when in when a passenger in cars/taxis/mini-buses the next 4 weeks	**1**	**2**	**3**	**4**	**5**	**6**	**7**

**Get a good night's sleep (more than 7 hours):**

**I get a good night's sleep (more than 7 hours) because … **	**Not true at all**			**Very true**
… I enjoy it when I manage to get a good night's sleep	**1**	**2**	**3**	**4**
… I value the benefits of getting a good night's sleep	**1**	**2**	**3**	**4**
… I will feel guilty if I do not get a good night's sleep	**1**	**2**	**3**	**4**
… people I know well (e.g., friends, parents etc.) say I should get a good night's sleep	**1**	**2**	**3**	**4**
… it is fun to get a good night's sleep	**1**	**2**	**3**	**4**
… .I think it is important to make the effort to get a good night's sleep	**1**	**2**	**3**	**4**
… I will feel ashamed if I do not get a good night's sleep	**1**	**2**	**3**	**4**
… I feel under pressure to get a good night's sleep from people I know well (e.g., friends, parents etc.)	**1**	**2**	**3**	**4**

**Table:**

**Read the statements below and circle the number on the right that best describes your answer**	**Strongly Disagree**						**Strongly agree**
I intend to get a good night's sleep in the next 4 weeks	**1**	**2**	**3**	**4**	**5**	**6**	**7**
I plan to get a good night's sleep in the next 4 weeks	**1**	**2**	**3**	**4**	**5**	**6**	**7**

**Keeping alcohol intake within guideline limits:**

**I keep my alcohol intake within the guideline limits for the number of units per day because … (N.B. guideline limits are 3 units for women = 2 small glasses of wine, 4 units for men = 2 pints of ordinary-strength beer)**	**Not true at all**			**Very true**
… I enjoy keeping my alcohol intake within the guideline limits of 3/4 units per day	**1**	**2**	**3**	**4**
… I value the benefits of keeping my alcohol intake within the guideline limits of 3/4 units per day	**1**	**2**	**3**	**4**
… I will feel guilty if I do not keep my alcohol intake within the guideline limits of 3/4 units per day	**1**	**2**	**3**	**4**
… people I know well (e.g., friends, parents etc.) say I should keep my alcohol intake within the guideline limits of 3/4 units per day	**1**	**2**	**3**	**4**
… it is fun to keep my alcohol intake within the guideline limits of 3/4 units per day	**1**	**2**	**3**	**4**
… .I think it is important to keep my alcohol intake within the guideline limits of 3/4 units per day	**1**	**2**	**3**	**4**
… I will feel ashamed if I don't keep my alcohol intake within the guideline limits of 3/4 units per day	**1**	**2**	**3**	**4**
… I feel under pressure to keep my alcohol intake within the guideline limits of 3/4 units per day from people I know well (e.g., friends, parents etc)	**1**	**2**	**3**	**4**

**Table:**

**Read the statements below and circle the number on the right that best describes your answer**	**Strongly Disagree**						**Strongly agree**
I intend to keep my alcohol intake within the guideline limits for number of units per day (3 for women, 4 for men) in the next 4 weeks	**1**	**2**	**3**	**4**	**5**	**6**	**7**
I plan to keep my alcohol intake within the guideline limits for number of units per day (3 for women, 4 for men) in the next 4 weeks	**1**	**2**	**3**	**4**	**5**	**6**	**7**

**Using condoms when having sexual intercourse:**

**I use condoms when having sex because … **	**Not true at all**			**Very true**
… I enjoy using condoms when having sex	**1**	**2**	**3**	**4**
… I value the benefits of using condoms when having sex	**1**	**2**	**3**	**4**
… I will feel guilty if I do not use condoms when having sex	**1**	**2**	**3**	**4**
… people I know well (e.g., sexual partner etc.) encourage me to use condoms when having sex	**1**	**2**	**3**	**4**
… it is fun to use condoms when having sex	**1**	**2**	**3**	**4**
… .I think it is important to make the effort to use condoms when having sex	**1**	**2**	**3**	**4**
… I will feel ashamed if I do not use condoms when having sex	**1**	**2**	**3**	**4**
… I feel under pressure to use condoms when having sex from the people I know well (e.g., sexual partner etc.)	**1**	**2**	**3**	**4**

**Table:**

**Read the statements below and circle the number on the right that best describes your answer**	**Strongly Disagree**						**Strongly agree**
I intend to use condoms on every occasion I have sex in the next 4 weeks	**1**	**2**	**3**	**4**	**5**	**6**	**7**
I plan to use condoms on every occasion I have sex in the next 4 weeks	**1**	**2**	**3**	**4**	**5**	**6**	**7**

**Wash my hands before preparing and handling food:**

**I wash my hands before preparing and handling food because … **	**Not true at all**			**Very true**
… I enjoy washing my hands before preparing and handling food	**1**	**2**	**3**	**4**
… I value the benefits of washing my hands before preparing and handling food	**1**	**2**	**3**	**4**
… I will feel guilty if I do not wash my hands before preparing and handling food	**1**	**2**	**3**	**4**
… people I know well (e.g., friends, parents etc.) say I should wash my hands before preparing and handling food	**1**	**2**	**3**	**4**
… it is fun to wash my hands before preparing and handling food	**1**	**2**	**3**	**4**
… .I think it is important to make the effort to wash my hands before preparing and handling food	**1**	**2**	**3**	**4**
… I will feel ashamed if I do not wash my hands before preparing and handling food	**1**	**2**	**3**	**4**
… I feel under pressure to wash my hands before preparing and handling food from people I know well (e.g., friends, parents etc.)	**1**	**2**	**3**	**4**

**Table:**

**Read the statements below and circle the number on the right that best describes your answer**	**Strongly Disagree**						**Strongly agree**
I intend to wash my hands before preparing and handling food in the next 4 weeks	**1**	**2**	**3**	**4**	**5**	**6**	**7**
I plan to wash my hands before preparing and handling food in the next 4 weeks	**1**	**2**	**3**	**4**	**5**	**6**	**7**

**Taking walks to relax and unwind:**

**I take walks to relax and unwind because … **	**Not true at all**			**Very true**
… I enjoy taking walks to relax and unwind	**1**	**2**	**3**	**4**
… I value the benefits of taking walks to relax and unwind	**1**	**2**	**3**	**4**
… I will feel guilty if I do not take walks to relax and unwind	**1**	**2**	**3**	**4**
… people I know well (e.g., friends, parents etc.) say I should take walks to relax and unwind	**1**	**2**	**3**	**4**
… it is fun to take walks to relax and unwind	**1**	**2**	**3**	**4**
… .I think it is important to make the effort to take walks to relax and unwind	**1**	**2**	**3**	**4**
… I will feel ashamed if I do not take walks to relax and unwind	**1**	**2**	**3**	**4**
… I feel under pressure to take walks walks to relax and unwind from people I know well (e.g., friends, parents etc.)	**1**	**2**	**3**	**4**

**Table:**

**Read the statements below and circle the number on the right that best describes your answer**	**Strongly Disagree**						**Strongly agree**
I intend to take walks to relax and unwind in the next 4 weeks	**1**	**2**	**3**	**4**	**5**	**6**	**7**
I plan to take walks to relax and unwind in the next 4 weeks	**1**	**2**	**3**	**4**	**5**	**6**	**7**

**Brushing your teeth:**

**I brush my teeth every day because … **	**Not true at all**			**Very true**
… I enjoy brushing my teeth every day	**1**	**2**	**3**	**4**
… I value the benefits of brushing my teeth every day	**1**	**2**	**3**	**4**
… I will feel guilty if I do not brush my teeth every day	**1**	**2**	**3**	**4**
… people I know well (e.g., friends, parents etc.) say I should brush my teeth every day	**1**	**2**	**3**	**4**
… it is fun to brush my teeth every day	**1**	**2**	**3**	**4**
… .I think it is important to make the effort to brush my teeth every day	**1**	**2**	**3**	**4**
… I will feel ashamed if I do not brush my teeth every day	**1**	**2**	**3**	**4**
… I feel under pressure to brush my teeth every day from people I know well (e.g., friends, parents etc.)	**1**	**2**	**3**	**4**

**Table:**

**Read the statements below and circle the number on the right that best describes your answer**	**Strongly Disagree**						**Strongly agree**
I intend to brush my teeth every day in the next 4 weeks	**1**	**2**	**3**	**4**	**5**	**6**	**7**
I plan to brush my teeth every day in the next 4 weeks	**1**	**2**	**3**	**4**	**5**	**6**	**7**

**Avoiding eating ‘junk’ food:**

**I avoid eating ‘junk’ food because … **	**Not true at all**			**Very true**
… I enjoy avoiding eating ‘junk’ food	**1**	**2**	**3**	**4**
… I value the benefits avoiding eating ‘junk’ food	**1**	**2**	**3**	**4**
… I will feel guilty if I eat ‘junk’ food	**1**	**2**	**3**	**4**
… people I know well (e.g., friends, parents etc.) say I should avoid eating ‘junk’ food	**1**	**2**	**3**	**4**
… it is fun to avoid eating ‘junk’ food	**1**	**2**	**3**	**4**
… .I think it is important to make the effort to avoid eating ‘junk’ food regularly	**1**	**2**	**3**	**4**
… I will feel ashamed if I eat ‘junk’ food	**1**	**2**	**3**	**4**
… I feel under pressure to avoid eating ‘junk’ food from people I know well (e.g., friends, parents etc.)	**1**	**2**	**3**	**4**

**Table:**

**Read the statements below and circle the number on the right that best describes your answer**	**Strongly Disagree**						**Strongly agree**
I intend to avoid eating ‘junk’ food in the next 4 weeks	**1**	**2**	**3**	**4**	**5**	**6**	**7**
I plan to avoid eating ‘junk’ food in the next 4 weeks	**1**	**2**	**3**	**4**	**5**	**6**	**7**

**Reducing consumption of caffeine:**

**I reduce my intake of caffeine because … **	**Not true at all**			**Very true**
… I enjoy reducing my consumption of caffeine	**1**	**2**	**3**	**4**
… I value the benefits of reducing my consumption of caffeine	**1**	**2**	**3**	**4**
… I will feel guilty if I consume caffeine	**1**	**2**	**3**	**4**
… people I know well (e.g., friends, parents etc.) say I should reduce my consumption of caffeine	**1**	**2**	**3**	**4**
… it is fun to reduce my consumption of caffeine	**1**	**2**	**3**	**4**
… .I think it is important to reduce my consumption of caffeine	**1**	**2**	**3**	**4**
… I will feel ashamed if I consume caffeine	**1**	**2**	**3**	**4**
… I feel under pressure to reduce consuming caffeine from people I know well (e.g., friends, parents etc.)	**1**	**2**	**3**	**4**

**Table:**

**Read the statements below and circle the number on the right that best describes your answer**	**Strongly Disagree**						**Strongly agree**
I intend to reduce consuming caffeine in the next 4 weeks	**1**	**2**	**3**	**4**	**5**	**6**	**7**
I plan to reduce consuming caffeine in the next 4 weeks	**1**	**2**	**3**	**4**	**5**	**6**	**7**

**Using the stairs instead of an elevator or escalator:**

**I use the stairs instead of an elevator or escalator because … **	**Not true at all**			**Very true**
… I enjoy using the stairs instead of an elevator or escalator	**1**	**2**	**3**	**4**
… I value the benefits of using the stairs instead of an elevator or escalator	**1**	**2**	**3**	**4**
… I will feel guilty if I do not using the stairs instead of an elevator or escalator	**1**	**2**	**3**	**4**
… people I know well (e.g., friends, parents etc.) say I should use the stairs instead of an elevator or escalator	**1**	**2**	**3**	**4**
… it is fun to go use the stairs instead of an elevator or escalator	**1**	**2**	**3**	**4**
… .I think it is important to make the effort to use the stairs instead of an elevator or escalator regularly	**1**	**2**	**3**	**4**
… I will feel ashamed if I do not use the stairs instead of an elevator or escalator	**1**	**2**	**3**	**4**
… I feel under pressure to use the stairs instead of an elevator or escalator from people I know well (e.g., friends, parents etc.)	**1**	**2**	**3**	**4**

**Table:**

**Read the statements below and circle the number on the right that best describes your answer**	**Strongly Disagree**						**Strongly agree**
I intend to use stairs instead of an elevator or escalator in the next 4 weeks	**1**	**2**	**3**	**4**	**5**	**6**	**7**
I plan to use stairs instead of an elevator or escalator in the next 4 weeks	**1**	**2**	**3**	**4**	**5**	**6**	**7**

**Wash my hands after going to the toilet:**

**I wash my hands after going to the toilet because … **	**Not true at all**			**Very true**
… I enjoy washing my hands after going to the toilet	**1**	**2**	**3**	**4**
… I value the benefits of washing my hands after going to the toilet	**1**	**2**	**3**	**4**
… I will feel guilty if I do not wash my hands after going to the toilet	**1**	**2**	**3**	**4**
… people I know well (e.g., friends, parents etc.) say I should wash my hands after going to the toilet	**1**	**2**	**3**	**4**
… it is fun to wash my hands after going to the toilet	**1**	**2**	**3**	**4**
… .I think it is important to make the effort to wash my hands after going to the toilet	**1**	**2**	**3**	**4**
… I will feel ashamed if I do not wash my hands after going to the toilet	**1**	**2**	**3**	**4**
… I feel under pressure to wash my hands after going to the toilet from people I know well (e.g., friends, parents etc.)	**1**	**2**	**3**	**4**

**Table:**

**Read the statements below and circle the number on the right that best describes your answer**	**Not true at all**						**Very true**
I intend to wash my hands after going to the toilet in the next 4 weeks	**1**	**2**	**3**	**4**	**5**	**6**	**7**
I plan to wash my hands after going to the toilet in the next 4 weeks	**1**	**2**	**3**	**4**	**5**	**6**	**7**

**Take dietary supplements to maintain a healthy diet (e.g., vitamin tablets, diet products, protein powders):**

**Take dietary supplements to maintain a healthy diet because … **	**Not true at all**			**Very true**
… I enjoy taking dietary supplements to maintain a healthy diet	**1**	**2**	**3**	**4**
… I value the benefits of taking dietary supplements to maintain a healthy diet	**1**	**2**	**3**	**4**
… I will feel guilty if I do not take dietary supplements to maintain a healthy diet	**1**	**2**	**3**	**4**
… people I know well (e.g., friends, parents etc.) say I should take dietary supplements to maintain a healthy diet	**1**	**2**	**3**	**4**
… it is fun to take dietary supplements to maintain a healthy diet	**1**	**2**	**3**	**4**
… .I think it is important to make the effort to take dietary supplements to maintain a healthy diet	**1**	**2**	**3**	**4**
… I will feel ashamed if I do not take dietary supplements to maintain a healthy diet	**1**	**2**	**3**	**4**
… I feel under pressure to take dietary supplements to maintain a healthy diet from people I know well (e.g., friends, parents etc.)	**1**	**2**	**3**	**4**

**Table:**

**Read the statements below and circle the number on the right that best describes your answer**	**Strongly Disagree**						**Strongly agree**
I intend to take dietary supplements to maintain a healthy diet in the next 4 weeks	**1**	**2**	**3**	**4**	**5**	**6**	**7**
I plan to take dietary supplements to maintain a healthy diet in the next 4 weeks	**1**	**2**	**3**	**4**	**5**	**6**	**7**

**Exercise regularly for more than 30 minutes at a time:**

**I exercise regularly for more than 20 minutes at a time because … **	**Not true at all**			**Very true**
… I enjoy exercising regularly	**1**	**2**	**3**	**4**
… I value the benefits of exercising regularly	**1**	**2**	**3**	**4**
… I will feel guilty if I do not exercise regularly	**1**	**2**	**3**	**4**
… people I know well (e.g., friends, parents etc.) say I should exercise regularly	**1**	**2**	**3**	**4**
… it is fun to exercise regularly	**1**	**2**	**3**	**4**
… .I think it is important to make the effort to exercise regularly	**1**	**2**	**3**	**4**
… I will feel ashamed if I do not exercise regularly	**1**	**2**	**3**	**4**
… I feel under pressure to exercise regularly from people I know well (e.g., friends, parents etc.)	**1**	**2**	**3**	**4**

**Table:**

**Read the statements below and circle the number on the right that best describes your answer**	**Strongly Disagree**						**Strongly agree**
I intend to exercise regularly (3-4 times per week) in the next 4 weeks	**1**	**2**	**3**	**4**	**5**	**6**	**7**
I plan to exercise regularly (3-4 times per week) in the next 4 weeks	**1**	**2**	**3**	**4**	**5**	**6**	**7**

**Plan work in advance to reduce deadline pressure and stress:**

**I plan work in advance to reduce deadline pressure and stress because … **	**Not true at all**			**Very true**
… I enjoy planning work in advance to reduce stress	**1**	**2**	**3**	**4**
… I value the benefits of planning work in advance to reduce stress	**1**	**2**	**3**	**4**
… I will feel guilty if I do not plan work in advance to reduce stress	**1**	**2**	**3**	**4**
… people I know well (e.g., friends, parents etc.) say I should plan work in advance to reduce stress	**1**	**2**	**3**	**4**
… it is fun to plan work in advance to reduce stress	**1**	**2**	**3**	**4**
… .I think it is important to make the effort to plan work in advance to reduce stress	**1**	**2**	**3**	**4**
… I will feel ashamed if I do not plan work in advance to reduce stress	**1**	**2**	**3**	**4**
… I feel under pressure to plan work in advance to reduce stress from people I know well (e.g., friends, parents etc.)	**1**	**2**	**3**	**4**

**Table:**

**Read the statements below and circle the number on the right that best describes your answer**	**Strongly Disagree**						**Strongly agree**
I intend to plan all my work in advance to reduce stress in the next 4 weeks	**1**	**2**	**3**	**4**	**5**	**6**	**7**
I plan to plan all my work in advance to reduce stress in the next 4 weeks	**1**	**2**	**3**	**4**	**5**	**6**	**7**

**Sitting with correct posture to avoid back pain:**

**I sit with correct posture to avoid back pain because … **	**Not true at all**			**Very true**
… I enjoy sitting with correct posture to avoid back pain	**1**	**2**	**3**	**4**
… I value the benefits of sitting with correct posture to avoid back pain	**1**	**2**	**3**	**4**
… I will feel guilty if I do not sit with correct posture to avoid back pain	**1**	**2**	**3**	**4**
… people I know well (e.g., friends, parents etc.) say I should sit with correct posture to avoid back pain	**1**	**2**	**3**	**4**
… it is fun to sit with correct posture to avoid back pain	**1**	**2**	**3**	**4**
… .I think it is important to make the effort to sit with correct posture to avoid back pain regularly	**1**	**2**	**3**	**4**
… I will feel ashamed if I do not sit with correct posture to avoid back pain	**1**	**2**	**3**	**4**
… I feel under pressure to sit with correct posture to avoid back pain from people I know well (e.g., friends, parents etc.)	**1**	**2**	**3**	**4**

**Table:**

**Read the statements below and circle the number on the right that best describes your answer**	**Strongly Disagree**						**Strongly agree**
I intend to sit with correct posture to avoid back pain in the next 4 weeks	**1**	**2**	**3**	**4**	**5**	**6**	**7**
I plan to sit with correct posture to avoid back pain in the next 4 weeks	**1**	**2**	**3**	**4**	**5**	**6**	**7**

**Avoiding foods high in sodium/salt (e.g., salted, pickled or smoked products):**

**I avoid foods high in sodium/salt (e.g., salted, pickled or smoked products) because … **	**Not true at all**			**Very true**
… I enjoy avoiding foods high in sodium/salt	**1**	**2**	**3**	**4**
… I value the benefits avoiding foods high in sodium/salt	**1**	**2**	**3**	**4**
… I will feel guilty if I do not avoid foods high in sodium/salt	**1**	**2**	**3**	**4**
… people I know well (e.g., friends, parents etc.) say I should avoid foods high in sodium/salt	**1**	**2**	**3**	**4**
… it is fun to avoid foods high in sodium/salt	**1**	**2**	**3**	**4**
… .I think it is important to make the effort to avoid foods high in sodium/salt	**1**	**2**	**3**	**4**
… I will feel ashamed if I do not avoid foods high in sodium/salt	**1**	**2**	**3**	**4**
… I feel under pressure to avoid foods high in sodium/salt from people I know well (e.g., friends, parents etc.)	**1**	**2**	**3**	**4**

**Table:**

**Read the statements below and circle the number on the right that best describes your answer**	**Strongly Disagree**						**Strongly agree**
I intend to avoid foods high in sodium/salt in the next 4 weeks	**1**	**2**	**3**	**4**	**5**	**6**	**7**
I plan to avoid foods high in sodium/salt in the next 4 weeks	**1**	**2**	**3**	**4**	**5**	**6**	**7**

**Eating foods containing sufficient dietary fibre (roughage) (e.g., wholegrain cereals and bread, fruit and vegetables):**

**I eat foods containing sufficient dietary fibre (roughage) (e.g., wholegrain cereals and bread, fruit and vegetables) because … **	**Not true at all**			**Very true**
… I enjoy eating foods containing sufficient dietary fibre	**1**	**2**	**3**	**4**
… I value the benefits of eating foods containing sufficient dietary fibre	**1**	**2**	**3**	**4**
… I will feel guilty if I do not eat foods containing sufficient dietary fibre	**1**	**2**	**3**	**4**
… people I know well (e.g., friends, parents etc.) say I should eat foods containing sufficient dietary fibre	**1**	**2**	**3**	**4**
… it is fun to eat foods containing sufficient dietary fibre	**1**	**2**	**3**	**4**
… .I think it is important to make the effort to eat foods containing sufficient dietary fibre	**1**	**2**	**3**	**4**
… I will feel ashamed if I do not eat foods containing sufficient dietary fibre	**1**	**2**	**3**	**4**
… I feel under pressure to eat foods containing sufficient dietary fibre from people I know well (e.g., friends, parents)	**1**	**2**	**3**	**4**

**Table:**

**Read the statements below and circle the number on the right that best describes your answer**	**Strongly Disagree**						**Strongly agree**
I intend to eat foods containing sufficient dietary fibre in the next 4 weeks	**1**	**2**	**3**	**4**	**5**	**6**	**7**
I plan to eat foods containing sufficient dietary fibre in the next 4 weeks	**1**	**2**	**3**	**4**	**5**	**6**	**7**

**Eating fruit/vegetables:**

**I eat 5 portions of fruit and vegetables per day because … **	**Not true at all**			**Very true**
… I enjoy eating 5 portions of fruit and vegetables per day	**1**	**2**	**3**	**4**
… I value the benefits of eating 5 portions of fruit and vegetables per day	**1**	**2**	**3**	**4**
… I will feel guilty if I do not eat 5 portions of fruit and vegetables per day	**1**	**2**	**3**	**4**
… people I know well (e.g., friends, parents etc.) say I should eat 5 portions of fruit and vegetables per day	**1**	**2**	**3**	**4**
… it is fun to eat 5 portions of fruit and vegetables per day	**1**	**2**	**3**	**4**
… .I think it is important to make the effort to eat 5 portions of fruit and vegetables regularly	**1**	**2**	**3**	**4**
… I will feel ashamed if I do not eat 5 portions of fruit and vegetables per day	**1**	**2**	**3**	**4**
… I feel under pressure to eat 5 portions of fruit and vegetables per day from people I know well (e.g., friends, parents etc.)	**1**	**2**	**3**	**4**

**Table:**

**Read the statements below and circle the number on the right that best describes your answer**	**Strongly Disagree**						**Strongly agree**
I intend to eat 5 portions of fruit and vegetables per day in the next 4 weeks	**1**	**2**	**3**	**4**	**5**	**6**	**7**
I plan to eat 5 portions of fruit and vegetables per day in the next 4 weeks	**1**	**2**	**3**	**4**	**5**	**6**	**7**

**PART 2: WHAT YOU ARE LIKE**

In this section, please read the pairs of statements, one pair at a time, and think about which statement within the pair seems more true for you. Indicate the degree to which statement A feels true, relative to the degree that Statement B feels true, on the 5-point scale shown after each pair of statements. If statement A feels completely true and statement B feels completely untrue, the appropriate response would be 1. If the two statements are equally true, the appropriate response would be a 3. If only statement B feels true, the appropriate response would be a 5; and so on.

**FOLLOW-UP (TIME 2) QUESTIONNAIRE**

**YOUR OPINIONS ABOUT YOUR EVERYDAY PASS TIMES AND BEHAVIOURS**

Thank you for agreeing to participate in the second part of our survey which asks your ***opinions*** about your participation in *everyday pass times* and *behaviours* in the ***past four**weeks***. Everyone does things differently so there are no right or wrong answers, we are interested what you ***actually do***. Do not spend too long on any one statement and give the response that best describes your feelings. All responses are strictly ***confidential***, and please answer ***all the questions*.**
